# 4104 Development and validation of parental empathy analog tasks

**DOI:** 10.1017/cts.2020.380

**Published:** 2020-07-29

**Authors:** Samantha Gonzalez, Christina Rodriguez

**Affiliations:** 1 University of Alabama at Birmingham

## Abstract

OBJECTIVES/GOALS: Parents’ empathy toward their children affects their parenting, which can in turn impact child outcomes. Although parental empathy is theoretically distinct from trait empathy, current literature relies on largely self-report measures of parents’ trait empathy. Thus, the current study evaluated new analog assessments of parental empathy. METHODS/STUDY POPULATION: One parental empathy analog measure (Empathy Measure for Parents Analog Task, Emotion Script; EMPAT-ES) was created based on parents’ responses to open ended prompts describing scenarios that elicit different emotions (e.g., happy, mad, sad, scared) in children. These responses were used to create short scripts. A second analog task (EMPAT, Emotion Audio) was created using 20 sec audio clips of children expressing the different emotions wherein participants respond with how they feel hearing the emotions and separately, how they believe the child feels. After an initial pilot, both versions of the EMPAT-E were administered to 120 families enrolled in a prospective longitudinal study. Parents completed self-report measures of trait empathy and parental empathy, as well as the EMPAT-ES and EMPAT-EA analog tasks. RESULTS/ANTICIPATED RESULTS: Internal consistency of both the EMPAT-ES and EMPAT-EA tasks are expected to be robust, demonstrating the reliability of these novel assessments of parental empathy. Results are also expected to demonstrate the construct and convergent validity of both analog tasks. These new measures of parental empathy are expected to be significantly associated with measures of trait empathy. Specifically, parents’ responses indicating how they believe the child feels in the analog are expected to be strongly related to their reported emotion recognition abilities and responses indicating how analog items made parents feel are expected to be related to parents’ empathic concern. Finally, parents’ responses to the analog tasks are anticipated to be strongly associated with parents’ self-reported parental empathy. DISCUSSION/SIGNIFICANCE OF IMPACT: Valid, novel assessments of parental empathy can impact the parenting literature as well as community intervention and prevention efforts with parents. Such analog tasks can bolster parenting research but they may also be translated to the community setting as a training tool wherein parents are taught new skills that promote more positive parenting.

